# Sensitization to Staphylococcus Enterotoxin: Relationship with Aspects of Disease Severity

**DOI:** 10.3390/jcm13195836

**Published:** 2024-09-30

**Authors:** Pinelopi Schoini, Vasiliki Apollonatou, Maria Kallieri, Myrto Blizou, Maria Sfika, Nektarios Koufopoulos, Abraham Pouliakis, Emmanouil Liatsis, Periklis Foukas, Petros Bakakos, Stelios Loukides

**Affiliations:** 1 ICU, Elpis Hospital, 115 22 Athens, Greece; pschoini@yahoo.com; 2 2nd Respiratory Department, Attikon University Hospital, National and Kapodistrian University of Athens, 124 62 Athens, Greece; vicky_apoll@hotmail.com (V.A.); mkallieri@yahoo.gr (M.K.); myrto_bl@hotmail.com (M.B.); maria.sfka@gmail.com (M.S.); 3 2nd Department of Pathology, Attikon University Hospital, National and Kapodistrian University of Athens, 124 62 Athens, Greece; koufonektar@gmail.com (N.K.); apou1967@gmail.com (A.P.); pfoukas@yahoo.com (P.F.); 4 Immune Laboratory Department, Agia Sophia Paediatric Hospital, 115 27 Athens, Greece; manolisliatsis@gmail.com; 5 1st Respiratory Department, Sotiria Hospital, National and Kapodistrian University of Athens, 115 27 Athens, Greece; petros44@hotmail.com

**Keywords:** severe asthma, endotypes, inflammation, *Staphylococcus aureus* enterotoxins, remodeling

## Abstract

**Background/Objective**: Sensitization to specific IgE *Staphylococcus aureus* enterotoxins (SEs) is associated with an increased risk for severe asthma development. Limited data exist regarding the association of seropositivity for specific IgE SEs and the different aspects of severe asthma. We aimed to determine whether the presence of SEs is associated with asthma-related parameters such as inflammatory cells in the airways, features of airway remodeling, and other variables relating to asthma assessment and severity. **Methods**: Fifty patients with severe asthma were recruited in the study. Demographics, comorbidities, asthma duration, and asthma medication were recorded by treating physicians. Specific IgE SE measurement, lung function, atopic status, asthma control test (ACT), sputum induction, bronchoscopy with BAL, and indices of airway remodeling were also assessed. **Results**: Twelve patients were positive to enterotoxin sensitization. Patients seropositive to specific IgE SEs significantly differed in regard to FEV_1_% pred and FEV_1_/FVC ratio compared to seronegative ones. Analyzing the inflammatory variables obtained from induced sputum, BAL, and endobronchial biopsies, the only significant difference was that of smooth muscle area (SMA), which was greater in specific IgE SE seropositive patients. The multivariate linear regression analysis showed two significant associations of specific IgE SE seropositivity. We found a negative with FEV_1_% pred with beta standardized coefficient 95%CI −0.054 (−0.083, −0.031), *p* < 0.001, and a positive with SMA with beta standardized coefficient 95%CI 0.054 (0.081, 0.037), *p* < 0.001. **Conclusions**: Seropositivity to specific IgE SEs in severe asthma is associated with more severe airflow limitation, obstruction, and upregulation in SMA, indicating a possible role in the remodeling process.

## 1. Introduction

Asthma is a heterogeneous disease characterized by different phenotypes and endotypes. Severe asthma is a unique phenotype of the disease, characterized by two major endotyping mechanisms—the high T2 process, characterized by increased production of IL-4, IL-5, IL-13, and high tissue densities of eosinophils and mast cells, and the low T2 process, characterized by lack of eosinophils and type 2 biomarkers. The definition of severe asthma is mainly based on the high requirements of treatment regimens and the limited control of the disease [1]. Different parameters obtained from different techniques or assessed by clinical and functional aspects drive the pathophysiology of severe asthma [2]. Airway remodeling is a major hallmark of asthma. Considering the high heterogeneity of the disease, airway remodeling may contribute to the disease process in a variable way, since many aspects may affect its major characteristics [3]. However, the assessment of airway remodeling relies on biopsy sampling. Remodeling characteristics include epithelial damage and cilial dysfunction, goblet cell hyperplasia, increased thickness of the lamina reticularis and reticular basement membrane, increased vascularity, and increased airway smooth muscle mass. The strongest predictor of airway limitation is excessive airway smooth muscle mass [4].

*Staphylococcus aureus* is a non-motile, Gram-positive, catalase-positive microbe. Around 20–25% of population is persistently colonized by *S. aureus*, and 60% are intermittent carriers [5]. *Staphylococcus aureus* can synthesize coagulases and a vast variety of toxins, such as toxic shock syndrome toxin-1 (TSST-1) and enterotoxin types A, B, C, through U, which are potent T-cell superantigens [6]. Many studies have addressed the association between severe asthma and the presence of specific IgE *Staphylococcus aureus* enterotoxins (SEs) [7]. Furthermore, published evidence supports that *Staphylococcus aureus* and its proteins are inducers of persistent type 2 airway inflammation [8]. They may further aggravate the control of the disease, the need for oral steroid use, the risk of hospitalization, and the impairment of lung function [9,10]. 

Considering the role of specific IgE SEs in severe asthma, we aimed to determine whether the presence of SEs is associated with inflammatory cells in the airways obtained either by sputum and/or by bronchoalveolar lavage (BAL), features of airway remodeling obtained by bronchial biopsies, and other different parameters relating to asthma assessment and severity, such as lung function, atopic status, and ACT score.

## 2. Materials and Methods

### 2.1. Patients

Patients were recruited from two respiratory departments: the University Respiratory Department in Sotiria Hospital, and the University Respiratory Department in Attikon University Hospital from October 2021 to April 2023. Fifty patients were included in the study. The diagnosis of severe asthma (SRA) was based on the ERS/ATS criteria [1]. The main inclusion criterion was severe asthma optimally treated. Subjects with any other respiratory disease or any concomitant malignant, heart, renal, liver, or collagen disease, as well as subjects with a respiratory tract infection or asthma exacerbation in the 8 weeks prior to study entry, were excluded. Demographics, comorbidities, asthma duration, and asthma medication were recorded by treating physicians. According to our regulatory ethics rules, the study was registered and approved at Sotiria Hospital (30777/14-12-2019). All participants provided written informed consent.

### 2.2. Lung Function

Forced Expiratory Volume in 1st sec (FEV_1_) and Forced Vital Capacity (FVC) were measured according to the ERS/ATS guidelines using two different spirometers [11]. All values were post-bronchodilation (PB) and expressed as % predicted.

### 2.3. Atopic Status

A positive skin prick test (mean wheal diameter of 3 mm or greater) to any of twenty common aeroallergens (including mites, grasses, trees, fungus, domestic animals) guided by clinical symptoms was used to confirm atopy. Other atopic diseases, such as atopic dermatitis, allergic rhinitis, and eczema, were also recorded. Total IgE (iu/mL) was also measured with the ImmunoCAP Total IgE (ThermoFisher Scientific, Branchburg, NJ, USA) fully quantitative test, which provides accurate levels of circulating IgE within a wide measuring range, between 2 and 5000 kU/L. Data were retrieved from patients’ medical records.

### 2.4. Enterotoxin Measurement

Baseline serum was analyzed for specific IgE SEA and SEB levels, using an ImmunoCAP 250 analyzer (Thermo Fisher, Uppsala, Sweden) according to the manufacturer’s instructions. SEA and SEB levels were classified into two groups: negative (both enterotoxins were <0.35 kU/L) and positive (either both enterotoxins were >0.35 kU/L or even one >0.35 kU/L). In some studies, SEA IgE detection levels was set at 0.1 kU/L [7]. Analysis took place at Pharmacology Laboratories of Athens Medical School and at Immunopathology Laboratories of “Agia Sofia” Paediatric Hospital.

Approximately 20 cc of blood was collected from each patient and gathered in 5 mL BD Vacutainer^®^ SST^®^ (Liuyang, China) tubes at room temperature. Tubes were centrifuged at 1000–1300 g (rcf) at room temperature for 10 min. After centrifugation, the supernatant was collected and stored at −80 °C until analysis.

### 2.5. Asthma Control Test (ACT)

ACT was assessed using the validated questionnaire, as previously described [12].

### 2.6. Sputum Induction

Sputum induction was performed according to a previously described methodology [13,14], using all the appropriate safety modifications, according to the underlying asthma severity. A sample was considered adequate when the patient was able to expectorate at least 2 mL of sputum, and the slides contained <10% squamous cells on differential cell counting. The sample was divided into two portions. The first one was processed using selected plugs, as previously described, for inflammatory cell identification [15]. Dithiothreitol (DTT) was added in a volume equal to four times the weight of the sputum specimen and it was further diluted with phosphate buffered saline (PBS) in a volume equal to the sputum plus DTT. At least 500 inflammatory cells were counted in each sample. Total cell count was expressed as the number of cells ×10^6^. Sputum inflammatory cells were expressed as (%) differential counts and as the absolute count (×10^6^/mL). Sputum samples were tested for inflammatory cell identification and slides were prepared for differential cell counting.

### 2.7. Bronchoscopy and Airway Remodeling Assessment

Bronchoscopy was performed on an outpatient basis according to published recommendations in all included patients, based on their availability to perform the procedure [16]. Bronchoalveolar lavage (BAL) was performed according to the European Respiratory Society Task Force guidelines [17]. Endobronchial biopsy specimens were obtained following BAL collection, from various sites of the subsegmental carinae of the right upper lobe, right lower lobe, and left upper lobe. Six bronchial biopsy specimens were obtained from each subject, fixed in formalin, and embedded in paraffin blocks before cutting.

Staining was performed on 4 μm deparaffinized sections. Two histochemical stains, i.e., Hematoxylin and Eosin (H&E) and Periodic Acid Schiff (PAS) (Artisan Link Pro auto stainer [DAKO]), and one immunostaining with anti-Smooth Muscle Actin (a-SMA) antibody were used for the evaluation of the integrity of the surface epithelium, the measurement of the basement membrane thickness, and the percentage of the smooth muscle area per bronchial biopsy, respectively.

Primary antibody against Alpha-Smooth Muscle Actin (mouse monoclonal, clone 1A4, DAΚO, Santa Clara, CA, USA) was used and immunohistochemistry was performed using the DAKOautostainer Link 48 device with EnVisionTMFLEX detection and visualization kit (K8002 [DAKO]). For antigen retrieval, slides were immersed in a high-pH solution (K8004 [DAKO]) and boiled in the PT Link Module (DAKO) for 20 min at 97 °C and subsequently cooled at 65 °C. Prior to mounting, all sections were immersed in Envision Flex wash buffer (K8007 [DAKO]) for 5 min and lightly counterstained with Harris Hematoxylin for 45 s.

Image analysis was performed using whole slide images (WSIs). Three histological slides per patient (H&E, PAS, and a-SMA immunostaining) were scanned using a Menarini D-Sight Fluo 200 slide scanner (Menarini Diagnostics S.r.l., Firenze, Italy) using 20× objective. In the sequel, virtual slides were converted from Jpeg 2000 format to TIFF and enabled porting to Fiji image analysis software (version 1.54f, a successor of the popular image analysis platform ImageJ). Special software was developed using the scripting language supported by Image J to create a user interface (the NKUA slide processor); this software enabled automated (batch) processing of virtual slides to perform measurements on multiple WSIs. In more detail, virtual slides were sliced into tiles with dimensions of 4000 × 4000 pixels, as it was not possible to process them as single images (due to their size); each tile was separately processed, and the results were aggregated to represent the entire virtual slide measurements. Integrity of the epithelium was defined as the percentage of length of the basement membrane with intact epithelium [18]. Reticular basement membrane thickness was measured only in well-orientated epithelial areas suitable for assessment. We performed repeated measures at regular intervals of 50 μm, in a way similar to that suggested by Sullivan et al. [19]. Reticular basement membrane thickness was calculated as the mean of at least 20 measures per case. Smooth muscle was calculated as the percentage of the area stained with SMA wth respect to the whole area of the biopsy [20].

### 2.8. Study Design

On day 1, all subjects underwent medical history and physical examination by an experienced pneumonologist. Demographics and ACT were recorded, and spirometry was performed for measuring FEV_1_, FVC. A detailed assessment of medical records was performed, and medical information relating to co-morbidities and atopy status was retrieved. On the same day, blood was drawn for the measurement of specific IgE SEs. The following day, sputum induction was performed. Around 10–15 days after the sputum induction, bronchoscopy was performed on an outpatient basis for each patient.

## 3. Statistical Analysis

Normally distributed data are presented as mean ± standard deviation (SD), whereas skewed data are presented as median and interquartile range [IR]. Normality of distribution was checked with Kolmogorov–Smirnov test. Differences in numerical variables between two groups were evaluated with unpaired *t*-tests and Mann–Whitney U-tests for normally and skewed data, respectively, whereas comparisons of proportions were performed using chi-square tests. To examine the associations between the presence of enterotoxins and the various parameters, a multivariate linear regression analysis was performed in one model, involving all severe asthmatic patients, and using the presence of specific IgE SEs as the dependent variable. All linear regressions were performed using age, gender, body mass index, atopy, co-morbidities, duration of the disease, and treatment regimens as covariates. Data were interpreted as standardized coefficients with 95% confidence intervals. A *p*-value < 0.05 was considered significant. Statistical analysis was performed using Medcalc (software Ostend Belgium, Version 9.3.7.0) and Graph pad prism 5 (GraphPad Software, San Diego, CA, USA).

## 4. Results

**Figure 1:**
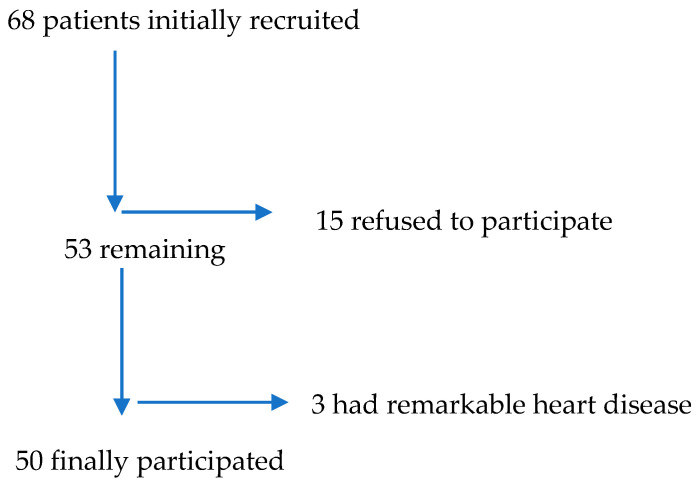
Flow chart of recruited patients.

We initially screened 68 severe asthmatics, and we finally recruited 50 (15 refused to participate, while the remaining three had remarkable heart disease). A flow chart is provided in Figure 1. Subjects’ characteristics are provided in Table 1.

Sensitization to specific IgE SEs was positive in twelve patients (eleven in both enterotoxins and one in SEA). Demographic characteristics of seronegative and seropositive patients are provided in Table 1.

**Figure 2:**
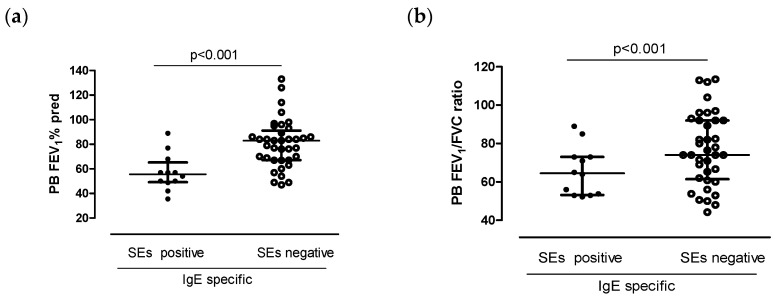
(**a**) Post-bronchodilation FEV_1_% predicted in patients with specific IgE SE positive and specific IgE SE negative, respectively. (**b**) Post-bronchodilation FEV_1_/FVC ratio in patients with specific IgE SE positive and specific IgE SE negative, respectively. Values are presented as median interquartile ranges. Statistically significantly lower in specific IgE SE positive. SE = staphylococcus enterotoxin.

Patients seropositive to SEs significantly differed in regard to both FEV_1_% pred and FEV_1_/FVC ratio compared to seronegative ones (Table 1, Figure 2a,b, *p* < 0.001).

**Figure 3:**
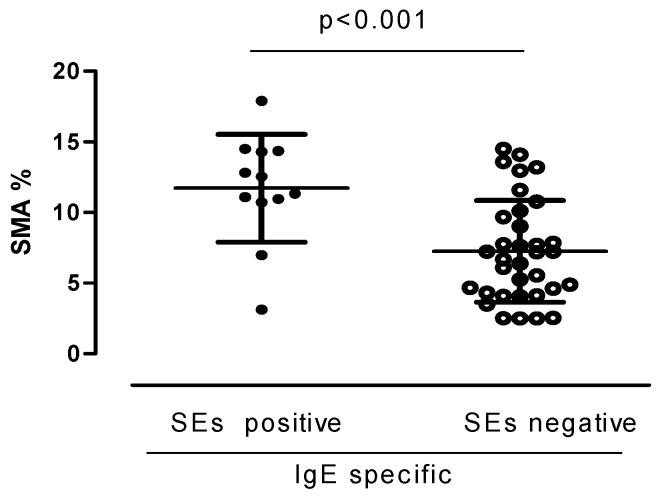
SMA% area in patients with specific IgE SE positive and SE negative, respectively. Values are presented as median interquartile ranges. Statistically significantly higher in specific IgE SE positive. SMA = smooth muscle area, SE = staphylococcus enterotoxin.

**Figure 4:**
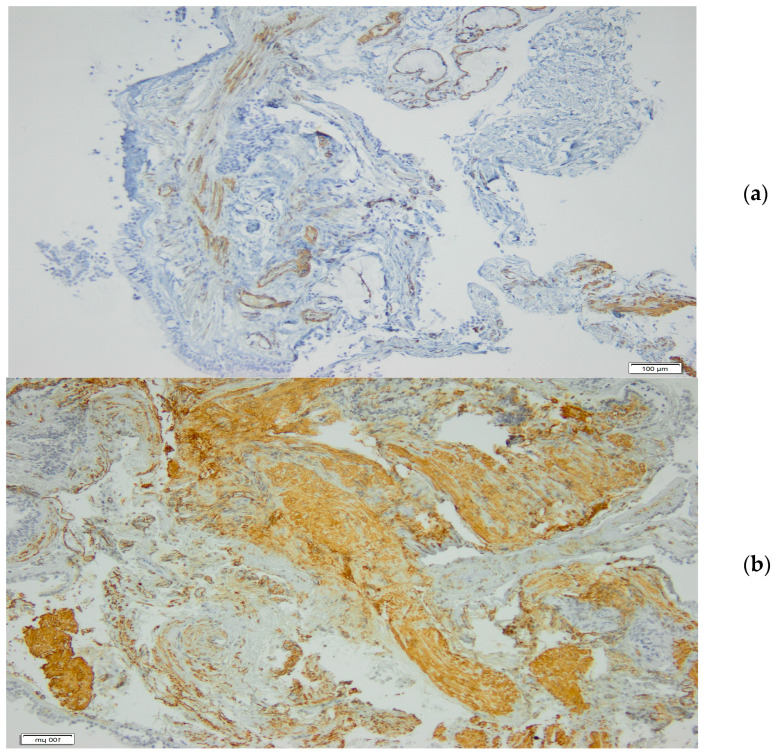
Representative images of bronchial biopsies from two patients with severe asthma, one seronegative (**a**) and one seropositive (**b**) to specific IgE SE, immunostained with anti-a-SMA (brown area). SMA = smooth muscle area, SE = staphylococcus enterotoxin.

Analyzing the variables related to inflammation, obtained from induced sputum and BAL, no differences were found between specific IgE SE seropositive and seronegative patients. Interestingly, the analysis of the histological parameters of remodeling showed a significantly increased SMA in specific IgE SE seropositive patients (*p* < 0.01) (Table 2, Figure 3). Representative images of bronchial biopsies from two patients with severe asthma, one seronegative (a) and one seropositive (b) to SE, are provided in Figure 4. The remaining variables were not significantly different.

The multivariate linear regression analysis showed two significant associations. A negative with FEV_1_% pred with beta standardized coefficient 95%CI −0.054 (−0.083, −0.031), *p* < 0.001, and a positive with SMA with beta standardized coefficient 95%CI 0.054 (0.081, 0.037), *p* < 0.001. When we excluded the patients with seropositivity to SEA-IgE, the aforementioned results did not change.

We re-analyzed our data according to a cut-off level of 0.1 kU/L. Only three patients were added in the positive group. All comparisons and the regression analysis did not change the already presented results.

## 5. Discussion

In this prospective observational study, we showed that patients with severe asthma and positive serum specific IgE to staphylococcal enterotoxins had increased SMA and more severe airflow limitation and obstruction compared to seronegative ones.

There is clear evidence that *Staphylococcus aureus* enterotoxin sensitization is associated with either the prevalence and/or the risk of asthma [7]. Furthermore, many studies support the notion that severe asthma is a major determinant in the above association [21]. The degree of sensitization varied among the studies [10,22], with some of them reporting high prevalence for severe disease [21]. In our study, approximately 25% of the study participants with documented severe asthma were found to be positive to specific IgE SEs. The above percentage seems low compared to other studies. In the previous studies, and particularly in the longitudinal study in the EGEA cohort, the percentages ranged from 62 to 74% [21]. However, the characterization of groups refers to moderate to severe asthma and not exclusively to severe asthma. In a multicentric Italian cohort of severe asthmatic patients, the prevalence of SE-IgE sensitization was approximately 25%, similar to ours [22]. Furthermore, the low percentage may be attributed to the low number of participants. However, our study was not an epidemiological study but a mechanistic one.

Different aspects of severe asthma have been linked to the presence of specific IgE SEs. Among them are chronic rhinosinusitis with nasal polyps [22], female gender [22], late-onset disease [9], increased exacerbation rate [23], smoking status [24], atopic dermatitis [25], eczema [26], impaired lung function, and increased airway reversibility [27]. In our study, none of the above were associated with the presence of specific IgE SEs. The only exception was airflow limitation and airflow obstruction. However, the last variable was lost in the process of multiple regression, indicating that either it is not strong enough as an effect of specific IgE SEs, or it may have been affected by confounding factors. Our study consisted of patients with severe asthma, optimally treated, most of them treated with biologics, and well controlled according to their ACT score. We consider the above critical for the interpretation of the findings related to the specific IgE SEs. Expanding the above hypothesis, we speculate that any association observed in the previous studies between staphylococcus enterotoxins and different aspects of the disease might be attributed to the non-controlled asthma and the low prevalence of effective treatment strategies. This is partially supported by the subjects’ characteristics, provided in two recent studies [22,23], where both the control of the disease and the underlying treatment strategies are lacking sufficient data.

Impaired lung function has already been observed in previous studies [7,23]. In one of those studies, it was mainly associated with age [28]. Our study clearly demonstrates that airflow limitation and obstruction differed between seropositive and seronegative asthmatics. However, the only major determinant of the specific IgE SE seropositivity was that of airflow limitation. Many hypotheses exist for the above association, such as the release of mast cell mediators, which can promote airway narrowing [29]. Additionally, the release of IL-5 [30], the respective attraction of eosinophils, and the further release of extracellular taps and galectin-10 promote the forming of Charcot–Leyden crystals [31]. This process maintains the inflammatory response in the airways and possibly impacts the airway narrowing. Based on our prospective study, we propose, as a major factor of airflow limitation in specific IgE SE seropositive patients, the presence of increased airway smooth muscle mass. This structural change may be the aggravating factor that could explain the airflow limitation and obstruction observed in different studies—including our study—in patients with sensitization to specific IgE SEs. Interestingly, no other features of airway remodeling were related to seropositivity to enterotoxins. Airway wall remodeling that could account for the chronic airflow limitation might occur through the amplified effects of T2 immune response via the release of IL-33 [32]. IL-33 can directly affect the epithelium and indirectly promote either the proliferation of airway smooth muscle cells and/or subepithelial fibrosis.

The study consists of some limitations, which are mainly attributed to the number of participants. We decided not to determine cut-off thresholds, double positives, and double negatives, since, in our initial analysis, respective numbers are not representative for further statistical analysis. The number of sensitized subjects is not well matched to the number of non-sensitized subjects. However, this is consistent with other studies that have linked sensitization to asthma pathogenesis [33]. Finally, as we mentioned in the methods section, we used two different spirometers for the lung function measurements. However, forty-four patients performed lung function tests in one spirometer, and only six in the other. Summarizing our study, sensitization to specific IgE SEs may provide valuable mechanistic information in the field of airflow limitation/obstruction in severe asthma. Furthermore, it emphasizes that optimal treatment in severe asthmatics may diminish different aspects of disease severity. However, the above association does not necessarily mean a causal relationship between seropositivity and bronchial smooth muscle hypertrophy. More research should be conducted to comprehend our findings and possibly use them for therapeutic purposes.

## Figures and Tables

**Table 1:** Demographic characteristics.

Variables	SRA (n = 50)	Specific IgE SE (+) n = 12	Specific IgE SE (−) n = 38
Age	53 ± 12	51 ± 13	54 ± 11
Gender (female/male)	33/17	9/3	25/13
Atopy (n &%)	29/50–58%	8/12–66%	21/38–55%
Nasal Polyps (n &%)	17/50–34%	4/12–30%	13/38–34%
Atopic dermatitis (n &%)	5/50–10%	2/12–16%	3/38–8%
Eczema (n &%)	4/50–8%	1/12–8%	3/38–8%
Allergic Rhinitis (n &%)	24/50–48%	5/12–41%	19/38–50%
Duration of asthma (years)	17.4 ± 15.4	18.6 ± 15.5	16 ± 15
Smoking (current/ex/never)	3/13/34	1/4/8	2//9/17
BMI Kg/m^2^	24.7 (23, 27)	24.7 ± 3.9	25 (23, 27)
ACT	21 (19, 24)	20 (18, 23)	21 (19, 23)
PB FEV_1_% pred.	75 ± 21	55 (49, 65)	81 ± 20 *
PB FEV_1_/FVC%	73 ± 8	65 ± 12	77 ± 18 *
Treatment regimens			
ICS	50 ^	12 ^	35
LABA	48	11	37
CS per os	14^^	4 ^^^	10
LAMA	18	5	13
LTRA	16	5	11
Omalizumab	5	2	3
Mepolizumab	16	7	9
Benrlazumab	6	4	2

* Data are presented as mean ± standard deviation (SD) or as median (interquartile ranges) according to normality. Abbreviations: BMI = body mass index, PB FEV_1_ = forced expiratory volume in one second post-bronchodilation, FVC = forced vital capacity, ICS = inhaled corticosteroids, LABA = long-acting β2 agonist, CS = corticosteroid, LTRA: leukotriene receptor antagonist, LAMA = long-acting muscarinic antagonist, SRA = Severe Refractory Asthma, SE = staphylococcus enterotoxin. ^ >800 μg budesonide/day or equivalent. ^^ Ten were receiving 5 mg prednisolone/day, while two were receiving 7.5 mg prednisolone/day, two were receiving 10 mg prednisolone/day, and one was receiving 15 mg prednisolone per day. ^^^ Three were receiving 5 mg prednisolone/day and one was receiving 15 mg prednisolone per day. * Statistically significant difference with *p* < 0.001.

**Table 2:** Major inflammatory variables in BAL, IS, and endobronchial biopsies.

Variables	Specific IgE SE (+) n = 12	Specific IgE SE (−) n = 38	*p*-Value
Cells × 10^6^/mL BAL	3.2(2.1–4.7)	3.8 (2.4, 5.1)	0.37
Eosinophils (%) BAL	1.1 (0, 8)	1.3 (0, 5)	0.62
Neutrophils (%) BAL	2.4 (0.6, 6.9)	2 (1, 4)	0.81
Cells × 10^6^/mL IS	18 (14–21)	19 (15–21)	0.621
Eosinophils (%) IS	1 (0, 4)	1 (0–3.5)	0.77
Neutrophils (%) IS	25 (8, 52)	21 (11, 30)	0.55
Eosinophils (%) blood	4.4 (1.7, 8.5)	3.6 (2.5, 6.5)	0.38
Eosinophils AC blood	252 (155, 668)	204 (121, 560)	0.45
IgEiu/mL	176 (124, 288)	160 (114, 275)	0.66
SMA %	11.7 ± 3.8	7.2 ± 3.6	**<0.01**
BMT μm	9 ± 1.6	9.4 ± 1.22	0.67
Integrity of the epithelium %	40 ± 22	35 ± 25	0.65

Data are presented as mean ± standard deviation (SD) or median (interquartile ranges). Bold numbers indicate significant differences. *p*-values indicate differences between the 2 groups. Abbreviations: BAL= bronchoalveolar lavage, IS = induced sputum, SE = staphylococcus enterotoxin, SMA= smooth muscle area, BMT= basement membrane thickness.

## Data Availability

Data are contained within the article.

## Abbreviations

ACT	asthma control test
AERD	aspirin-exacerbated respiratory disease
APC	antigen-presenting cell
ATS	American Thoracic Society
BAL	bronchoalveolar lavage
BMI	body mass index
BMT	basement membrane thickness
CS	corticosteroid
DTT	dithiothreitol
ERS	European Respiratory Society
FEV_1_	forced expiratory volume in 1 s
FVC	Forced Vital Capacity
ICS	inhaled corticosteroid
H&E	Hematoxylin & Eosin
IgE	Immunoglobulin E
IL-5	interleukin-5
IL-4	interleukin-4
IL-13	interleukin-13
IL-33	interleukin-33
IL-25	interleukin-25
IS	induced sputum
LABA	long-acting β_2_ agonists
LAMA	long-acting muscarinic antagonists
LTRA	leukotriene receptor antagonists
PAS	Periodic Acid Schiff
PB	post-bronchodilation
RBM	reticularbasement membrane
RCF	Relative Centrifugal Force
SD	standard deviation
SEs	*Staphylococcus aureus* enterotoxins
SMA	smooth muscle area
SRA	Severe Refractory Asthma
TCRV*β*	TCR molecule
TSLP	thymic stromal lymphopoietin
TSST-1	toxic shock syndrome toxin-1
WSI	whole slide image
